# Severe Ventral Erosion of Penis Caused by Indwelling Urethral Catheter and Inflation of Foley Balloon in Urethra—Need to Create List of “Never Events in Spinal Cord Injury” in order to Prevent These Complications from Happening in Paraplegic and Tetraplegic Patients

**DOI:** 10.1155/2010/461539

**Published:** 2010-06-27

**Authors:** Subramanian Vaidyanathan, Bakul M. Soni, Peter L. Hughes, Gurpreet Singh, Tun Oo

**Affiliations:** ^1^Regional Spinal Injuries Centre, District General Hospital, Southport, Merseyside PR8 6PN, UK; ^2^Department of Radiology, District General Hospital, Southport, Merseyside PR8 6PN, UK; ^3^Department of Urology, District General Hospital, Southport, Merseyside PR8 6PN, UK

## Abstract

Never Events are serious, largely preventable patient safety incidents that should not occur if the available preventative measures have been implemented. We propose that a list of “Never Events” is created for spinal cord injury patients in order to improve the quality of care. To begin with, following two preventable complications related to management of neuropathic bladder may be included in this list of “Never Events.” (i) Severe ventral erosion of glans penis and penile shaft caused by indwelling urethral catheter; (ii) incorrect placement of a Foley catheter leading to inflation of Foley balloon in urethra. If a Never Event occurs, health professionals should report the incident through hospital risk management system to National Patient Safety Agency's Reporting and Learning System, communicate with the patient, family, and their carer as soon as possible about the incident, undertake a comprehensive root cause analysis of what went wrong, how, and why, and implement the changes that have been identified and agreed following the root cause analysis.

## 1. Introduction

Never Events are serious, largely preventable patient safety incidents that should not occur if the available preventative measures have been implemented. A policy on Never Events was introduced in the National Health Service in England from April 2009, following its proposal in High Quality Care for All [1]. The policy is designed to promote transparency and accountability when serious patient safety incidents occur. The National Patient Safety Agency was established in England 2001 with a mandate to identify patient safety issues and find appropriate solutions. The National Patient Safety Agency published a core list of eight Never Events for 2009/2010. The core list of Never Events the following.

- Wrong site surgery: a surgical intervention performed on the wrong site (e.g., wrong knee, wrong eye, wrong patient, wrong limb, or wrong organ).
- Retained instrument postoperation: one or more instruments or swabs, or a throat pack, are unintentionally retained following an operative procedure, and an operation or other invasive procedure is needed to remove this, and/or there are complications to the patient arising from its continued presence.
- Wrong route administration of chemotherapy: intravenous or other chemotherapy (e.g., vincristine) that is correctly but administered via the wrong route (usually into the intrathecal space).
- Misplaced nasogastric or orogastric tube not detected prior to use: naso or orogastric tube placed in the respiratory tract rather than the gastrointestinal tract and not detected prior to commencing feeding or other use.
- Inpatient suicide using noncollapsible rails.
- In-hospital maternal death from postpartum haemorrhage after elective caesarean section: in-hospital death of a mother as a result of a haemorrhage following elective caesarean section, excluding cases where imaging has identified placenta accreta.
- Intravenous administration of wrongly selected concentrated potassium chloride.
- Escape from within the secure perimeter of medium or high secure mental health services by patients who are transferred prisoners.

We propose that a list of “Never Events” is created for spinal cord injury patients in order to improve the quality of care. To begin with, following two preventable complications related to management of neuropathic bladder may be included in this list of “Never Events.”

- Severe ventral erosion of glans penis and shaft of penis caused by indwelling urethral catheter.
- Incorrect placement of a Foley catheter leading to inflation of Foley balloon in urethra.

Severe ventral erosion of penis relates only to those cases where the urethral catheter has cut through glans penis and shaft of penis as well. Patients with minor erosion of urethra, which is limited to glans penis such as wide-open urethra or fish mouth appearance, will not fall under the category of “Never Events.” Incorrect positioning of Foley catheter with balloon inflated in the urethra in a tetraplegic patient can lead to life-threatening autonomic dysreflexia, apart from causing bleeding from urethra and retention of urine in bladder. We present two cases to illustrate these complications, which should not have occurred if good care had been provided to spinal injury patients.

## 2. Case Presentation

### 2.1. Case 1—Severe Ventral Erosion of Penis Caused by Indwelling Urethral Catheter

**Figure 1:**
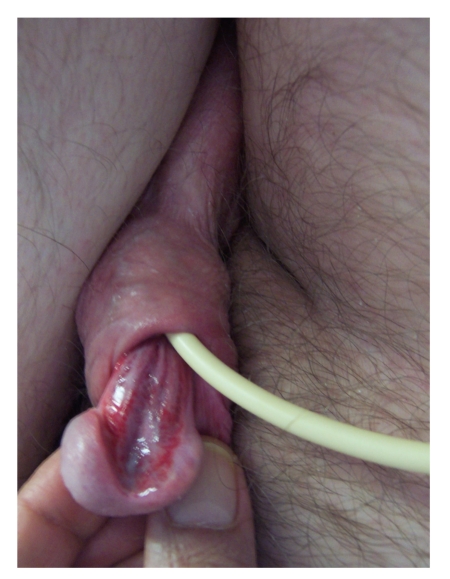
Clinical photograph of penis shows erosion of ventral surface of penis. The Foley catheter has eroded glans penis, penile skin and almost entire penile urethra.

A twenty-three-year-old male sustained C-5 incomplete tetraplegia in a road traffic accident in 1982. He underwent implantation of sacral anterior root stimulator in 1986. The receiver block was removed in 2007. He developed a 5 cm left trochanteric sore due to friction as he transferred himself from bed to chair. Considering this patient's condition, he was advised to have indwelling urethral catheter on 26 February 2009 in the community. In December 2009, this patient was admitted to spinal unit for management of pressure sore. Clinical examination revealed severe degree of erosion of urethra. Ventral erosion of penis was quite extensive and included glans penis and almost the entire penile urethra (Figure 1).

**Comments**

Erosion of urethra by an indwelling catheter is preventable. This patient was advised to manage his bladder by intermittent catheterisation. But intermittent catheterisations were not possible in the community. Therefore, this patient was left with long-term indwelling catheter, which resulted in erosion of urethra. Ventral erosion of penis by indwelling catheter can lead to bleeding from raw edges of split urethra, and increased chances of urine infection because of shorter urethra. Patients with urethral damage and erosion related to prolonged catheter present a formidable challenge in surgical reconstruction. Most have serious comorbidities and a single operation does not usually solve all the problems [2]. Casey and associates [3] studied eleven patients with neurogenic bladder dysfunction, who underwent urethral reconstruction. Men undergoing reconstruction for urethral erosion had inferior outcomes compared to those with other urethral pathology. Patients with spinal cord injury in whom urethral reconstruction is considered should be advised that urethral surgery carries a high risk of reoperation and eventual need for urinary diversion. Clearly, many patients with neurological disease and severe urethral pathology are best treated with urinary diversion [4].

The need for urinary diversion can be averted if spinal cord injury patients are not allowed to develop ventral erosion of penis by indwelling catheter. Anchoring the drainage tube of leg bag to thigh with a strap will allow free movement of catheter and avoid any pull on the catheter and penis. When the urethral catheter is fixed taut and the patient develops erection of penis, the indwelling catheter acts as a bowstring and cuts through the penis. Leaving the catheter slack prevents catheter-induced erosion of urethra especially when patient develops erection of penis. Of course, discarding indwelling urethral catheter is the best way to prevent erosion of urethra. Intermittent catheterisation is preferable to indwelling urinary catheter drainage. Suprapubic cystostomy would have prevented catheter-induced erosion of urethra.

### 2.2. Case 2—Inflation of Foley Balloon in Urethra

**Figure 2:**
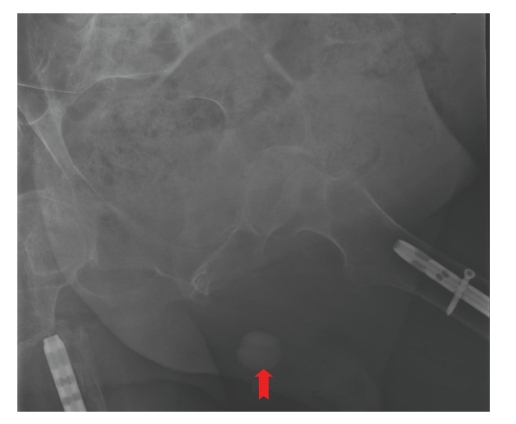
X-ray of pelvis was taken after injecting two mL of contrast through balloon channel of Foley catheter. The Foley balloon was located in scrotum (arrow) and not inside urinary bladder.

**Figure 3:**
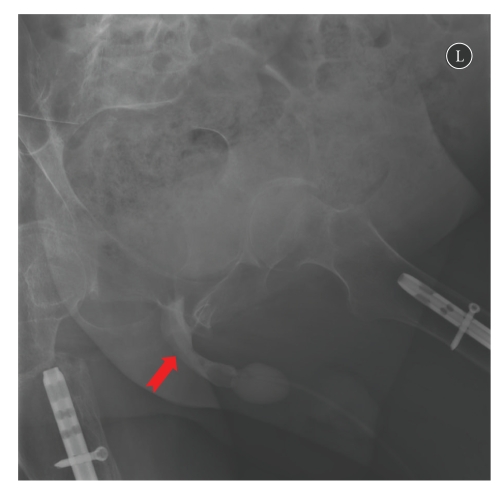
X-ray of pelvis: twenty mL of contrast was injected thorough lumen of Foley catheter. Proximal urethra was visualised by the injected contrast (arrow) thus confirming that the tip of Foley catheter was lying in urethra and not in the bladder. There was large amount of urine retained in the bladder.

**Figure 4:**
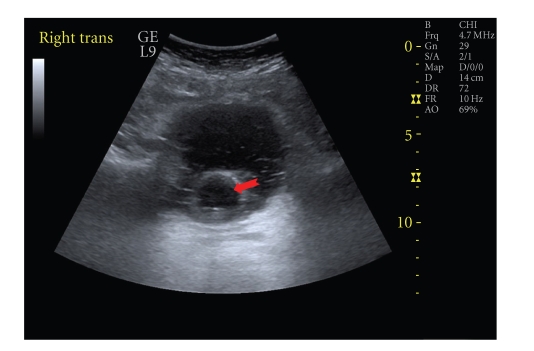
Follow-up ultrasound of urinary bladder after inserting a 16 French Foley catheter with 20 mL balloon. The balloon of Foley catheter was located within urinary bladder (arrow), thus confirming correct placement of Foley catheter.

A 56-year-old male with tetraplegia (C-5 incomplete) attended spinal unit in May 2009 with history of sweating. He started sweating after indwelling urethral catheter was changed by a community health professional. On examination, a long segment of Foley catheter was lying outside penis. The balloon of Foley catheter was palpable in the perineum. Clinical impression was that Foley catheter had been placed incorrectly with the balloon inflated in urethra. Five mL of water was aspirated from the balloon channel of Foley catheter. Then two mL of contrast (Optiray 300) was injected into the balloon channel of Foley catheter and X-rays were taken as per the radiological technique described for demonstration of incorrect positioning of Foley catheter [5]. X-ray of pelvis showed that balloon of Foley catheter was located in urethra and not inside urinary bladder (Figure 2). Then 20 mL of contrast was injected through mail lumen of Foley catheter and X-rays were taken. The contrast visualised the proximal penile urethra, thus confirming that the tip of Foley catheter was lying within urethra (Figure 3). The Foley balloon was deflated completely and then, Foley catheter was removed. A 16 French Foley catheter with 20 mL balloon was inserted. Follow-up ultrasound scan confirmed correct positioning of Foley balloon inside the urinary bladder (Figure 4).

**Comments**

Insertion of urinary catheter in a tetraplegic patient requires expert knowledge, skills and judgement. Spasm of urethral sphincter may hinder insertion of urethral catheter in a spinal cord injury patient. False passages in urethra if present, pose additional difficulties in urethral catheterisation. Even when a catheter is inserted into the bladder, sudden bladder spasm may push the catheter out before Foley balloon is inflated. In such a situation, an inexperienced health professional may not realise what has happened and unknowingly might inject water in to the balloon channel. This will lead to inflation of the balloon of Foley catheter in urethra, as indeed happened in this patient. If excessive length of Foley catheter lies outside penis, this indicates that “Long catheter sign” is positive and denotes incorrect placement of Foley catheter [6]. An astute health professional will spot this clinical sign but it may be elusive to a novice. This case illustrates that only senior health professionals should perform urethral catheterisation of a tetraplegic patient in order to minimise risks of catheter-related complications. Trainee nurses and intern doctors may not have gained sufficient expertise to carry out catheterisations in spinal cord injury patients.

Kashefi and associates from Division of Urology, University of California-San Diego School of Medicine, San Diego, California [7] recognised that iatrogenic urethral injuries were a substantial source of preventable morbidity in hospitalized male patients. These researchers designed and implemented a nursing education program that included basic urological anatomy, urethral catheter insertion techniques and catheter safety. Implementation of such a nursing education program significantly decreased the incidence of iatrogenic urethral injury and, thereby, improved patient safety.

## 3. Discussion

Severe ventral erosion of glans penis and shaft of penis caused by indwelling urethral catheter, and incorrect placement of Foley catheter in urethra are preventable complications. In a good spinal injuries centre these adverse events should never occur. Therefore, we wish to create a “Never Event List” for spinal cord injury patients. To begin with, following two preventable complications related to management of neuropathic bladder may be included in this list of “Never Events.”

- Severe ventral erosion of glans penis and penile shaft caused by indwelling urethral catheter.
- Incorrect placement of a Foley catheter leading to inflation of Foley balloon in urethra.

After spinal cord injury physicians have become familiar with the concept of “Never events”, and what to do when a never event occurs, this list should be expanded to include other adverse clinical incidents such as incorrect dosing of a pump implanted for intrathecal administration of baclofen in spinal cord injury patients.

We believe that spinal cord injury physicians should observe following protocol if a Never Event occurs.

- Report the incident through hospital risk management system to National Reporting and Learning Service, National Patient Safety Agency.
- Communicate with the patient or service user, family or their carer as soon as possible about the incident in line with the policy: http://www.npsa.nhs.uk/nrls/improvingpatientsafety/patient-safety-tools-and-guidance/beingopen/.
- Undertake a comprehensive root cause analysis of went wrong, how and why [8].
- National Patient Safety Agency's Incident Decision Tree may be used to decide what initial action to take with the staff involved in the incident [9]. This ensures a consistent and fair approach.
- Implement the changes that have been identified and agreed following the root cause analysis or significant event audit.
- Discuss the learning and corrective/preventative actions following the occurrence of the Never Event with all health professionals in spinal unit and in the community.

Following preliminary feedback from the National Health Service, initial data from the Reporting and Learning System of the National Patient Safety Agency, and recommendations in “The operating framework for the National Health Service in England 2010/11”, the Never Events framework outlines was updated with new aspects for 2010/11 [10]. The National Patient Safety Agency's National framework for reporting and learning from serious incidents, and Care Quality Commission registration require mandatory notification of serious events including “Never Events”. The operating framework for the National Health Service in England 2010/2011 reaffirmed its commitment to the following.

- Ensure that patient safety incidents which are Never Events are reported to the National Patient Safety Agency.
- Publish the numbers and types of events on an annual basis.

By applying this policy to spinal cord injury patients, it is likely that the quality of care will be is improved. Steps have already been taken by National Patient Safety Agency to decrease the risks of suprapubic catheter insertion [11], reduce harm from omitted and delayed medicines in hospital [12] and preventing complications due to inadvertent use of female urinary catheters in adult male patients [13]. A Never Event list addressing peculiar clinical problems encountered in spinal cord medicine is likely to improve patient care by increasing awareness amongst health professionals, who will be encouraged to identify and implement appropriate measures promptly in order to stop these complications from happening in spinal unit and in the community.
